# Structural and Physicochemical Characteristics of Oil Bodies from Hemp Seeds (*Cannabis sativa* L.)

**DOI:** 10.3390/foods10122930

**Published:** 2021-11-26

**Authors:** Francesca Louise Garcia, Sihan Ma, Anant Dave, Alejandra Acevedo-Fani

**Affiliations:** Riddet Institute, Massey University, Private Bag 11222, Palmerston North 4442, New Zealand; francescalouise.garcia@gmail.com (F.L.G.); S.Ma@massey.ac.nz (S.M.); anant.dave@fonterra.com (A.D.)

**Keywords:** hempseeds, physicochemical properties, oil bodies, oleosomes, structure, delivery systems

## Abstract

The structural and physicochemical characteristics of oil bodies from hemp seeds were explored in this study. Oil bodies from several plant-based sources have been previously studied; however, this is the first time a characterisation of oil bodies from the seeds of industrial hemp is provided. The morphology of oil bodies in hemp seeds and after extraction was investigated using cryo-scanning electron microscopy (cryo-SEM), and the interfacial characteristics of isolated oil bodies were studied by confocal laser scanning microscopy (CLSM). Proteins associated with oil bodies were characterised using sodium dodecyl sulphate polyacrylamide gel electrophoresis (SDS-PAGE). The effect of pH and ionic strength on colloidal properties of the oil bodies was investigated. Oil bodies in hemp seeds appeared spherical and sporadically distributed in the cell, with diameters of 3 to 5 μm. CLSM images of isolated oil bodies revealed the uniform distribution of phospholipids and proteins at their interface. Polyunsaturated fatty acids were predominant in the lipid fraction and linoleic acid accounted for ≈61% of the total fatty acids. The SDS-PAGE analysis of washed and purified oil bodies revealed major bands at 15 kDa and 50–25 kDa, which could be linked to membrane-specific proteins of oil bodies or extraneous proteins. The colloidal stability of oil bodies in different pH environments indicated that the isoelectric point was between pH 4 and 4.5, where oil bodies experienced maximum aggregation. Changes in the ionic strength decreased the interfacial charge density of oil bodies (ζ-potential), but it did not affect their mean particle size. This suggested that the steric hindrance provided by membrane-specific proteins at the interface of the oil bodies could have prevented them from flocculation at low interfacial charge density. The results of this study provide new tertiary knowledge on the structure, composition, and colloidal properties of oil bodies extracted from hemp seeds, which could be used as natural emulsions or lipid-based delivery systems for food products.

## 1. Introduction

Hemp (*Cannabis sativa* L.) cultivars with less than 0.3% or 0.2% of delta-9-tetrahydrocannabinol (THC) are actively grown for several industrial purposes including food applications. Production of hemp seeds from these varieties is rapidly increasing because of their great nutritional and functional value. The seeds from hemp are an excellent source of dietary lipid, protein, and fibre. The oil extracted from the hemp seeds contains more than 90% of polyunsaturated fatty acids (PUFAs) including two essential fatty acids (EFAs), which are linoleic acid and α-linolenic acid [1,2]. The high PUFA and EFA contents of hemp seed oil can improve health and development when included in the diet as EFAs cannot be synthesised by the human body. Moreover, hemp seed oil has an *ω*-6/*ω*-3 fatty acid ratio of around 2.5:1 that falls within the optimal balance of 2:1 and 3:1; this ratio is considered essential for the metabolism of PUFAs [1]. In addition, hemp seed oil contains bioactive compounds, such as tocopherols, flavonoids, chlorophyll pigments, and phenolic compounds [3]. Because of its high nutritive value, hemp seed oil shows great potential as a bioactive food ingredient. The oil is usually extracted by cold-pressing to preserve its bioactive compounds. Solvent extraction (usually carried out with hexane) is also used to increase the yield and reduce the cost of extraction [2]. However, this makes the oil inedible as it may result in oil degradation, having contaminants from residual solvents.

Within the seed, the oil is contained in small spherical intracellular organelles called oil bodies [4,5]. An oil body consists of a hydrophobic triacylglyceride (TAG) core surrounded by a complex membrane consisting of a monolayer of phospholipids embedded with proteins, called oleosins, that play a role in their synthesis and stability [5,6]. Among different species, the size of seed oil bodies varies but falls within a narrow range of 0.5–2.5 μm [4,7]. This narrow size range provides a large surface area for the attachment of lipase during seed germination for rapid mobilisation of reserve TAGs [5]. The isoelectric point of oil bodies generally ranges from 5.7 to 6.6 (which is close to the isoelectric point of oleosin), indicating that they have a net negative charge at their surface at neutral pH [7,8]. This is due to the interaction of the negatively charged phospholipids with the positively charged residues of the surface proteins [7]. The variation in the isoelectric point of oil bodies from different species may be attributed to the differences in the extraction process used for isolation since the chemicals used for extraction can remove the non-specifically associated proteins on the oil body surface [9,10,11,12].

Oil bodies are present in abundant amounts in plant tissues and can be considered as natural oil-in-water emulsions because of their unique structure [13,14]. The presence of oleosin at the interface provides oil bodies with steric hindrance, protecting the phospholipids against the action of phospholipases and also protecting oil bodies from coalescence or aggregation [6]. Moreover, the interaction between phospholipids and oleosins at the interface gives oil bodies a negative surface charge that leads to electrostatic repulsions [15]. This unique interface allows oil bodies to maintain their individuality and have remarkable stability inside the cell where they are stored for long periods until they are utilised for germination [4,5]. 

Hemp seed oil bodies can be a highly valuable novel ingredient for the food industry because of their potential as natural emulsions or nature-assembled delivery systems of PUFAs, EFAs, and liposoluble bioactive compounds. To date, no studies have been reported on the extraction of oil bodies from hemp seeds. Since the properties of oil bodies after extraction vary from source to source, the characterisation of new materials is necessary before their applications in food products. This study provides for the first time an in-depth characterisation of oil bodies from hemp seeds. The microstructure, composition, and the colloidal stability of oil bodies under different pH and ionic strength conditions were investigated to gain better understanding of their functional performance in food-like environments.

## 2. Materials and Methods

### 2.1. Materials

Unless stated otherwise, all chemicals were purchased from Sigma-Aldrich Ltd. (St. Louis, MO, USA), and the reagents were made up in Milli-Q water (Milli-Q apparatus; Millipore Corp., Bedford, MA, USA). Low-THC hulled hemp seed kernels (Supplier: Sunshine (Tianjin) Produce Limited, Country of Origin: China, AAA Grade, heat-treate) were purchased from a local supermarket (Davis Trading Company Ltd., Palmerston North, New Zealand). The content of THC of hemp seeds was less than 10 ppm, as declared by the supplier. The THC content in hemp seeds for food applications must be less than 0.35%, regulated by the Ministry of Health and New Zealand Legislation. All experiments were repeated at least twice and the means of the results from two or more experiments are reported.

### 2.2. Methods

#### 2.2.1. Preparation of Hemp Milk and Isolation of Oil Bodies

Dehulled hemp seeds were soaked overnight in Milli-Q water at a seed-to-water ratio of 1:4 (*w*/*w*). Then, the mixture was ground using a colloid mill at a frequency of 30 Hz (Bematek Systems, Inc., Model CZ-60-PB, Salem, MA, USA). To remove the solids, we passed the resulting mixture through a clarifier separator (GEA Westfalia, Model CTC-03-107, New Jersey, NJ, USA) and the “hemp milk” was collected for cream extraction. 

Hemp seed oil bodies were isolated from hemp milk by centrifugation at 10,000× *g* for 20 min at 20 °C. The resulting cream layer referred to as “oil bodies” or “oil body fraction” was then carefully removed from the aqueous layer for experimental studies and added with sodium azide (0.02% *w*/*w*) to inhibit the growth of microorganisms.

#### 2.2.2. Microstructure

The morphology of oil bodies in hemp seed and after isolation were examined by cryo-scanning electron microscopy (cryo-SEM), whereas the interfacial properties of the oil body fraction was determined by confocal laser scanning microscopy (CLSM).

##### Cryo-SEM

For the analysis of oil bodies in the cross-section of the seed, dehulled hemp seeds were placed in the sample holder and flash-frozen by dipping in liquid nitrogen. After 10 min of contact with liquid nitrogen, the frozen sample was transferred to the cryo-unit sectioning chamber and placed under vacuum. The temperature of the sample was lowered to −120 °C followed by fracturing using a cold knife.

For the sample imaging, the temperature of the sample was gradually raised to −100 °C for 20 min for sublimation. The fractured surface of the seeds was then coated with a thin platinum coating (10 mA for 240 s), and the samples were transferred to the imaging chamber. SEM images of fractured sections were recorded at 6 to 20 kV on a Joel JSM 6500F Field Emission Scanning Microscope. A total of five different sections were examined, and images were captured at different resolutions.

Similarly, the morphology of the hemp seed oil body fraction was examined by cryo-SEM as per the described method but following the sample preparation protocol of Efthymiou et al. [16]. Briefly, a drop of oil body suspension was placed between two small copper grids, and the excess sample was gently soaked dry by a filter paper. The sample sandwiched between the grids was then placed in a jet freezer assembly (Baltec JFD 030) and frozen rapidly to −186 °C using a continuous propane stream for 30 min. Upon freezing, the copper grid from one of the sides was removed, and the sample was placed on the sample holder maintained at −180 °C of the cryo-SEM apparatus and was transferred to the cryo-unit sectioning chamber and placed under vacuum. Sample imaging was performed as previously described with modifications. Instead of a platinum coating, the surface of the sample was coated with a thin palladium coating and SEM images of the fractured sections were recorded at 5 kV at −120 °C on a Joel JSM 6500F Field Emission Scanning Microscope equipped with an energy-dispersive detector and a Gatan Alto2500 cryo-unit.

##### Confocal Laser Scanning Microscopy (CLSM)

The microstructure of hemp seed oil bodies was investigated using a confocal laser scanning microscope (Model Leica SP5, DM6000B, supplied by Leica Microsystems, Heidelberg, Germany) with a 63 mm oil immersion objective lens. Nile Red (1 mg/mL in acetone) and Fast Green FCF (1 mg/mL in Milli-Q water) were used to selectively stain neutral lipids and proteins, respectively, on the basis of the staining protocols described by Gallier et al. [17]. Briefly, 200 μL of the diluted oil body fraction (0.1% *v*/*v* fat) was mixed with 12 μL of Nile Red and 6 μL of Fast Green FCF. The stained sample was placed on a concave microscope slide, covered with a cover slip (0.17 mm thick), and immediately examined using the confocal laser scanning microscope. For phospholipids staining, Lissamine™ rhodamine B dye (Rd-DHPE, 1 mg/mL in chloroform) was used. Briefly, 100 μL of the diluted oil body fraction (0.1% *v*/*v* fat) was added with 5 μL of Lissamine™ rhodamine B (Rd-DHPE). The stained sample was placed on a concave microscope slide, covered with a cover slip (0.17 mm thick), and examined using the confocal laser scanning microscope. The images were processed using the software ImageJ.

#### 2.2.3. Compositional Analysis of Hemp Seed Oil Bodies

##### Proximate Composition

The compositional analysis of the oil body fraction, such as moisture content, fat content, crude protein content, and ash content, was performed at the Nutrition Laboratory of Massey University, Palmerston North, New Zealand. The following methods were used for analysis: vacuum oven drying (AOAC 990.19, 990.20) for moisture, Mojonnier method (flour, baked, extruded products, AOAC 922.06) for fat content, Dumas method (AOAC 968.06) for crude protein content, and furnace drying at 550 °C (AOAC 942.05 for feed, meat) for ash content. 

##### Fatty Acid Composition

The total lipids from the hemp seed oil body fraction were extracted using the B&D method [18]. The neutral lipids and polar lipids were separated by solid phase extraction (SPE) as described by Avalli and Contarini [19]. In brief, 400 mg lipid sample was dissolved in 1 mL of chloroform–methanol (2:1, *v*/*v*), and 0.75 mL of the mixture was loaded into hexane conditioned HybridSPE^®^—Phospholipid cartridge (bed weight 30 mg, 1 mL, Sigma Aldrich Ltd., St. Louis, MO, USA). The neutral lipids were eluted with 3 mL of hexane–diethyl ether (8:2, *v*/*v*) and 3 mL of hexane–diethyl ether (1:1, *v*/*v*). The recovered fraction for neutral lipids was dried for further fatty acids analysis. The polar lipids were eluted with 4 mL of methanol and followed with 2 mL of methanol and 2 mL of chloroform–methanol–water (3:5:2, *v*/*v*/*v*). The recovered fraction for polar lipids was dried for further fatty acids analysis.

Total fatty acids analysis in both lipid fractions were quantified by gas chromatography (GC), following the method described by Zhu et al. [20]. A sample of 20 mg of lipid fraction (neutral or polar) and 1 mL methyl tricosanoate/hexane (1 mg/mL) (internal standard) were mixed. The composition of the total fatty acids was determined by capillary GC on a Supelcowax™ 10 (30 m × 0.35 mm × 0.50 lm) capillary column (Supelco Park, Bellefonte, PA, USA) installed in an Agilent 7890 gas chromatography system equipped with a flame ionisation detector and a split/splitless injector (Agilent Technologies, Santa Clara, CA, USA). The initial oven temperature was 140 °C, which was maintained for 1 min before being increased to 180 °C at a rate of 10 °C/min, and then maintained at 180 °C for 1 min. The oven temperature was then increased to 210 °C at a rate of 2 °C/min, and then maintained at 210 °C for 10 min. Helium was used as the carrier gas at 72 cm/s running at a constant flow. The injector was set at 250 °C, and the detector was set at 260 °C. The split ratio was 20:1. 

#### 2.2.4. Protein Composition of Hemp Seed Oil Bodies by Sodium Dodecyl Sulphate Polyacrylamide Gel Electrophoresis (SDS-PAGE)

The oil body fraction was collected by separating hemp milk at 10,000× *g* centrifugation for 20 min. The oil bodies were washed by two methods, and the final samples were referred to as “phosphate buffer-washed oil bodies” (WC) and “purified oil bodies” (POB), respectively. 

The oil body fraction was suspended in phosphate buffer (10 mM, pH 7.5) followed by centrifugation (10,000× *g* for 20 min). After washing it twice, the cream phase was collected and suspended with phosphate buffer, and then mixed with hexane (1:1) and re-centrifuged at 10,000× *g* for 20 min to remove free oil. The WC was suspended in phosphate buffer for analysis. 

The oil body fraction was purified by the method described by Tzen et al. [21]. Briefly, the oil body fraction was suspended in phosphate buffer (5 mM, pH 7.5) containing 0.1% (*w*/*w*) Tween 20 and 0.2 M sucrose. The suspension was diluted 1:1 using phosphate buffer (10 mM, pH 7.5) and centrifuged at 10,000× *g* for 20 min. The cream layer obtained was suspended in 9 M urea solution, and then the suspension was diluted 2:1 using phosphate buffer (10 mM, pH 7.5) followed by centrifugation at 10,000× *g* for 20 min. The cream phase was re-suspended in phosphate buffer (10 mM, pH 7.5) containing 0.6 M sucrose and washed by hexane as described above. The final POB was re-suspended in phosphate buffer for analysis. 

Sodium dodecyl sulphate polyacrylamide gel electrophoresis (SDS-PAGE) was performed under both non-reducing and reducing conditions as per the protocol described by Manderson et al. [22]. Hemp milk (M), phosphate buffer-oil bodies (WC), and purified oil bodies (POB) samples were mixed with PAGE sample reducing buffer and non-reducing buffer to a final protein concentration of 2 mg/mL. For the reducing condition, dithiothreitol was used in reducing sample buffer (200 mM), and the reducing samples were heated at 95 °C for 10 min. Then, 10–15 μL of samples were loaded onto Mini-Protean gels (Bio-Rad Laboratories, Richmond, CA, USA) and run at 120 V for about 120 min. The molecular imager Gel Doc XR system (Bio-Rad Laboratories, Richmond, CA, USA) was used for gel scanning, and ImageLab software was used for image analysis.

#### 2.2.5. Physicochemical Characterisation and Stability of Oil Bodies

##### Particle Size

The particle size of hemp seed oil bodies was measured by static light scattering using a Mastersizer 2000 (Hydro MU, Malvern, Worcestershire, UK). Samples were dispersed in Milli-Q water or a sodium dodecyl sulphate (SDS) solution (1.2% *w*/*v*). The data were reported in Sauter-average diameter (*d*_3,2_) and volume-mean diameter (*d*_4,3_). The refractive index of hemp seed oil (1.475) and water (1.33) was used in the protocol. The mean particle size was calculated as the average of triplicate measurements.

##### ζ-Potential

The ζ-potential of hemp seed oil bodies was determined using a Zetasizer Nano ZS (Malvern Instruments Ltd., Malvern, United Kingdom) equipped with a 4 mW He/Ne laser at a wavelength output of 633 nm. Samples were diluted to a final particle concentration of 0.01% (*v*/*v*) in Milli-Q water and put in an electrophoresis cell (Model DTS1070, Malvern Instruments Ltd.) at 25 °C. The ζ-potential was read at least 10 times for each sample, and the ζ-potential values were calculated by Smoluchowski approximation. Mean values were calculated from triplicate measurements.

##### Effect of pH and Ionic Strength on Colloidal Stability

The stability of oil bodies in the food matrix should be understood once they are incorporated in the food matrix of commercial products. Their stability may be affected by changes in their environment such as variations in pH, ionic strength, and/or temperature. To determine the influence of pH on the colloidal stability, we suspended hemp seed oil bodies in Milli-Q water at an oil body fraction-to-water ratio of 1:3 (*w*/*w*). The suspensions were adjusted to pH 3–10 using 0.1 M HCl or 0.1 M NaOH. For the effect of ionic strength, the hemp seed oil body fraction was suspended at different concentrations of NaCl (0, 62.5, 125, 250, 500, and 1000 mM). The particle size and ζ-potential of the samples were determined as described in ‘particle Size’ and ‘ζ-potential’ sections, respectively.

## 3. Results and Discussion

### 3.1. Morphology of Hemp Seed

**Figure 1:**
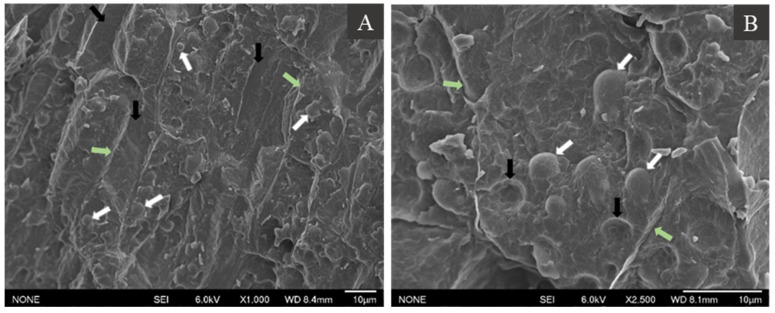
Cryo-SEM images of the cross-section of hemp seeds at 1000× (**A**) and 2500× (**B**) magnification. See the text for the description of the arrows.

Figure 1 shows the microstructure of hemp seed by Cryo-SEM. The hemp seeds were filled with oil bodies (shown in white arrows) that were sporadically distributed inside the cell (cell wall indicated in green arrows) (Figure 1). Oil bodies appeared spherical with diameters ranging from 3 to 5 μm with minor irregularities in the shape. Aside from the cell walls and the oil bodies, no other structural elements could be identified from the micrographs. Moreover, some cells seemed to be devoid of any oil bodies in the plane of imaging, as seen in Figure 1A (black arrows). In Figure 1B, oil bodies appeared to have an outer covering that may or may not be part of the oil body membrane or the seed membrane. In addition, some cells also showed semi-hemispherical cavities (black arrows, Figure 1B), which may have contained an oil body previously but were dislodged during fracturing of the sample. 

Although there are no reported studies on the microstructure of oil bodies in hemp seeds, other studies have examined the structural characteristics of other oleaginous seeds and tissues. For instance, oil bodies with an apparent diameter of 1 to 3 μm were found to be densely packed in *E. plantagineum* seeds [13]. The TEM images showed that oil bodies were sporadically distributed within the seed, with most cells being densely packed with oil bodies. More recently, Paterlini et al. [23] also reported the morphology of *Jathropha peiranoi* seeds, where the cells of endosperm and embryo tissues were rich in oil bodies, containing about 13–15 grouped spherical structures of 2.5–4.5 μm, occupying the cell volume. 

The dense cell environment causes the compression of oil bodies, leading to close packing [24]. The ability to withstand compression forces and maintain integrity indicates high flexibility of the hemp seed oil bodies as has been noted previously [25,26]. The close packing of oil bodies in the cell matrix also makes them appear asymmetrical. The same was observed for oil bodies in maize germ [25]. On the contrary, oil bodies in sunflower seeds have more spherical shape, partially attributed to the greater space available due to higher moisture content in the seeds [25]. 

The cryo-SEM images of hemp seeds also showed some cells with fewer oil bodies or completely devoid of any oil bodies (Figure 1). Such an asymmetric distribution of oil bodies has also been reported for coconut endosperm [26]. These regions in the cell without any oil bodies could be occupied by other intracellular organelles. It is possible that the images with asymmetric distribution of oil bodies represent a cross-section of cellular regions occupied by other intracellular organelles. 

### 3.2. Microstructure of Hemp Seed Oil Bodies

**Figure 2:**
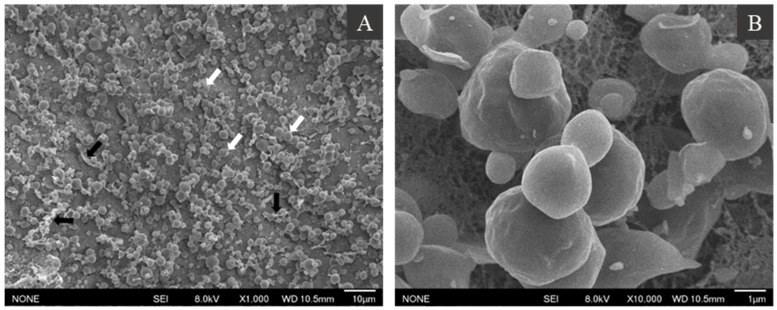
Cryo-SEM images of hemp seed oil bodies in an aqueous solution at 1000× (**A**) and 10,000× (**B**) magnification. For description of white and black arrows, please see text.

The morphology of isolated oil bodies in aqueous suspension was further examined through cryo-SEM imaging. Oil bodies appeared to be spherical in shape (white arrows, Figure 2A), with the diameter ranging from 2 to 5 μm (1000× magnification). The size of the oil bodies in suspension agreed with the size of oil bodies in the seed (Figure 1). At 10,000× magnification (Figure 2B), the oil bodies appeared to have an irregular spherical shape and a rough surface. Furthermore, thread-like structures or connections between oil droplets were observed (black arrows, Figure 2A), likely caused by artefacts arising during the sublimation of ice during sample preparation. 

**Figure 3:**
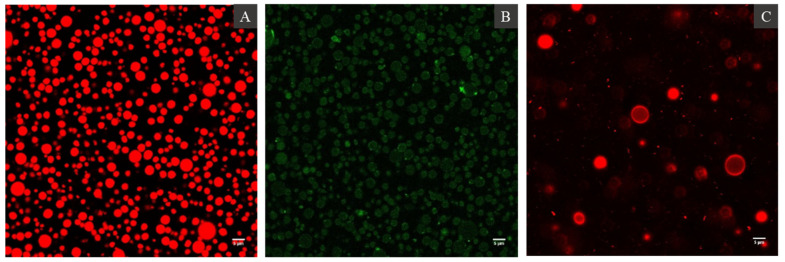
Confocal Laser Scanning Microcopy images of hemp seed oil bodies showing neutral lipids stained by Nile Red (**A**), proteins stained by Fast Green-FCF (**B**) dyes, and the interfacial distribution of phospholipids stained by Lissamine™ rhodamine B dye (**C**).

The microstructure of oil bodies was examined by confocal laser scanning microscopy using the dyes Nile Red, FG-FCF, and Lissamine™ rhodamine B (Rd-DHPE) to stain neutral lipids, proteins, and phospholipids, respectively. The CLSM images showed that hemp seed oil bodies existed as spherical droplets in aqueous solutions, and their diameter ranged from 3 to 7 μm without any noticeable flocculation or aggregation. They contained a large intense red fluorescent core region that represented the neutral lipids or TAGs (Figure 3A) surrounded by a uniform covering of proteins at the interface (stained green by FG-FCF), regardless of the oil body size (Figure 3B). No protein aggregates were observed in the continuous phase of the suspension. Lastly, staining with Rd-DHPE showed uniform intense red fluorescence on the surface of the oil droplets, confirming the presence of phospholipids (Figure 3B).

Both Cryo-SEM and CLSM imaging confirmed that oil bodies were not aggregated in aqueous suspension. No protein aggregates (stained by FG-FCF) were observed in the aqueous phase, indicating that the protocol was able to isolate intact oil bodies with minimal amounts of extraneous proteins. The hemp seed oil body interface appeared to have a uniform distribution of phospholipids as indicated by the intense red fluorescence on the oil body surface upon staining with Rd-DHPE. Overall, hemp seed oil bodies conform to the classic interfacial structure of seed bodies proposed by [5] and previously confirmed in oil bodies from nuts [17,27] and fruits [28] as well.

### 3.3. Proximate Composition of Hemp Seed Oil Bodies

The proximate composition of the hemp seed oil body fraction is shown in Table 1; this fraction had 79.3 ± 2.8% fat and 1.5 ± 0.2% protein, respectively. The protein content was within the range of 0.6 to 3.4% (*w*/*w*) reported for oil bodies from seeds of various species [7]. This could represent the proteins located at the oil body interface and possibly some extraneous proteins that were present in the oil body fraction after isolation. The phospholipid content in the oil body fraction was not determined in the study.

### 3.4. Fatty Acid Composition of Lipids in Hemp Seed Oil Bodies

Table 2 shows the fatty acid composition of the hemp seed oil body fraction. The composition of lipids in oil bodies contained ≥ 90% of unsaturated fatty acids composed mainly of long-chain fatty acids. Linoleic acid (C_18:2_) accounted for 61% of the total fatty acids. Other major fatty acids of lipids from hemp seed oil bodies were oleic acid (C_18:1_, 15.1%) and α-linolenic acid (C_18:3_, 11.7%). Saturated fatty acids such as palmitic acid (C_16:0_, 6.1%) and stearic acid (C_18:0_, 2.9%) were also found in minor concentrations. Teh and Birch [3], Oomah et al. [29], and Alonso-Esteban et al. [30] reported that linoleic acid was found as the major fatty acid in hemp seed oil (53–57%). The fatty acid composition found in oil bodies was similar to that previously reported for hemp oil. These similarities had been previously reported for other seeds and oil bodies fractions [13]. The *ω*-6/*ω*-3 ratio found in the oil body fraction was 2.6:1, which is in good aggrement with previous studies on oil extracted from hemp seed (2.5:1) [1]. The lipid composition of hemp seed oil can be comparable to walnut oil due to their high linoleic and α-linolenic acid content [31].

The composition of fatty acids in the neutral lipids fraction was very similar to the total lipids fraction of hemp seed oil bodies. This was expected because hemp seeds lipids mostly consist of TAG, which are neutral lipids, and about 1.5–2% are non-TAG lipids or unsaponifiable fraction [32]. Regarding the polar lipid fraction, the major fatty acids found were C_16:0_, C_18:1_, C_18:2_, and C_18:3_, following a similar trend to those in neutral lipids fraction but with higher levels of C_18:3_. 

Polar lipids are a diverse class of compounds that are divided into two main subclasses, phospholipids and sulfolipids [33]. Antonelli et al. [34] reported the polar lipid composition of hemp seed oil, and they found that 51% of the phospholipids were phosphatidylcholines (PC), and C_16:0_, C_16:1_, C_18:2_, and C_18:3_ were the most abundant fatty acid combinations. They also reported that about 72% of the sulfolipids were sulfoquinovosylmonoacylglycerols (SQDG), and the fatty acid combinations C_16:0_, C_18:1_, and C_18:3_ were the most abundant. Therefore, the major fatty acids found in the polar lipid fraction of hemp seed oil bodies are likely linked to the presence of PC and SQDG at high concentrations. 

### 3.5. Protein Composition

**Figure 4:**
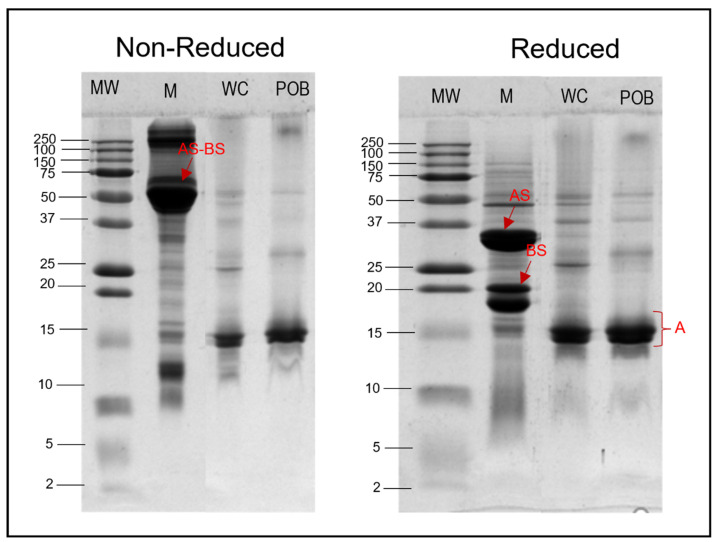
Composition of proteins in hemp seed oil bodies in reducing and non-reducing conditions. MW: molecular weight marker; M: hemp milk; WC: phosphate buffer-washed oil bodies; POB: purified oil bodies washed with urea.

The protein composition of the oil body fraction was determined using SDS-PAGE under reducing and non-reducing conditions (Figure 4). The gel patterns showed the polypeptide bands present in hemp milk (M), phosphate-washed oil bodies (WC), and purified oil bodies washed with urea (POB). 

The hemp milk (lane M) in both gels showed more than 30 protein bands ranging in MW from 250 to 5 kDa. The most intense bands identified were around MW 52 kDa (in non-reducing conditions) and MW 18, 20, and 34 kDa (under reducing conditions). These most likely corresponded to edestin, the major globulin of hemp seeds protein. Edestin is a hexamer with MW of about 300 kDa that is composed of six identical sub-units linked by non-covalent interactions, each unit having five cysteine residues [35,36,37]. Two of five cysteine residues were disulphide-linked through basic sub-units (BS) with MW about 18–20 kDa, and three acidic sub-units (AS) of MW around 34 kDa [38], which were visible in the gels made under reducing conditions (disulphide-linked Stibbards units are dissociated under these condition) (Figure 4). The polypeptide band at 52 kDa under non-reducing conditions could correspond to the AS-BS units linked by disulphide bonds. 

The number of polypeptide bands found in WC lane were significantly less than in M lane. Only around five bands were observed with molecular weight ranging from 55 to 25 kDa. These differences indicated that there are remarkably less proteins in the oil body fraction after isolation from hemp milk. The bands identified in WC lane may be linked to either membrane-specific proteins of oil bodies (oleosins, caleosins, steroleosins) [39], or to extraneous proteins carried over after isolation from hemp milk. Nikiforidis et al. [40] reported that oil bodies after isolation contain their own structural proteins (also called membrane-specific proteins) and exogenous proteins belonging to the matrix where oil bodies were isolated from, with these proteins varying from source to source. 

Similarly, the number of polypeptide bands found in POB lane were even less than those found in the WC lane (in reducing and non-reducing conditions), suggesting that proteins remaining in POB should be only those tightly linked to the membrane of oil bodies. The major band in POB at around 15 kDa could be related to oleosins. Maize germ oleosins have been previously identified by SDS-PAGE analysis, being located at 15 and 16 kDa bands [41]. Similarly, oleosins have been identified from oil bodies isolated from rice bran [42] and sesame seeds [43]. 

### 3.6. Physicochemical Properties of Hemp Seed Oil Bodies

#### 3.6.1. Particle Size and ζ-Potential

**Figure 5:**
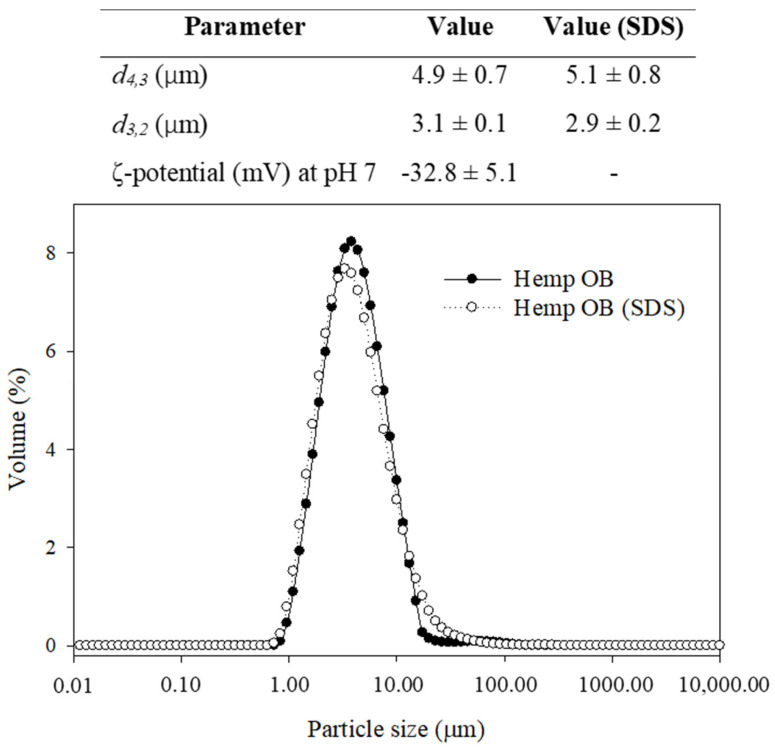
Particle size (*d*_4,3_ and *d*_3,2_) and ζ-potential of hemp seed oil bodies suspended in water and SDS.

The size distribution, average size, and charge of hemp seed oil bodies were characterised after dispersion in water or 1.2% SDS (Figure 5). The average diameter of oil bodies dispersed in water was *d*_4,3_: 4.9 ± 0.7 μm and *d*_3,2_: 3.1 ± 0.1 μm (Figure 5). These values agree with the size of oil bodies measured by cryo-SEM images (Figure 2) and CLSM images (Figure 3). This indicates that the integrity and structure of oil bodies in the seed are maintained upon extraction and that they exist as spherical droplets in solution. The size of oil bodies is higher than the range reported for other plant species which ranged from 0.5 to 2.5 μm; the size of oil bodies is generally influenced by biological factors [4,7]. Oil bodies that are smaller in size usually exhibit a low TAG to interfacial protein ratio, while those with larger sizes have lower interfacial protein content [4,40].

The presence of SDS in the dispersion did not cause a change in the *d*_4,3_ and *d*_3,2_ of oil bodies, which were 5.1 ± 0.8 μm and 2.9 ± 0.2 μm, respectively. SDS disrupts the hydrophobic interactions between membrane-specific proteins on the oil body surface and the extraneous proteins; thus, the mean particle diameter represented the real size of individual oil bodies [11]. Irrespective of the presence of SDS in the samples, the size distributions of the oil bodies showed a monomodal peak, indicating that most oil bodies had similar sizes. The similarities of size distributions of oil bodies with and without SDS also suggests the absence of flocculation or aggregation, which agrees with the results from CLSM imaging (Figure 3). The CLSM images showed the absence of protein aggregates (stained by FG-FCF) in the aqueous phase, suggesting that the protocol was able to isolate intact oil bodies with minimal amounts of extraneous proteins.

The ζ-potential of hemp seed oil bodies at pH 7 was found to be −32.8 ± 5.1 mV. The ζ-potential was measured at pH 7 in all samples to avoid fluctuations due to variations in the pH of hemp oil body extracts (results not shown). Along with the particle size results, the surface charge of oil bodies demonstrates their stability in aqueous suspension. At neutral pH, the oil bodies also exhibited a high magnitude of ζ-potential (absolute value above 30 mV), which indicates the good electrostatic stability of the oil bodies dispersed in water. A ζ-potential magnitude of 30 mV is an indication of good electrostatic stability of emulsions [44].

#### 3.6.2. Effect of pH and Ionic Strength on Colloidal Stability

**Figure 6:**
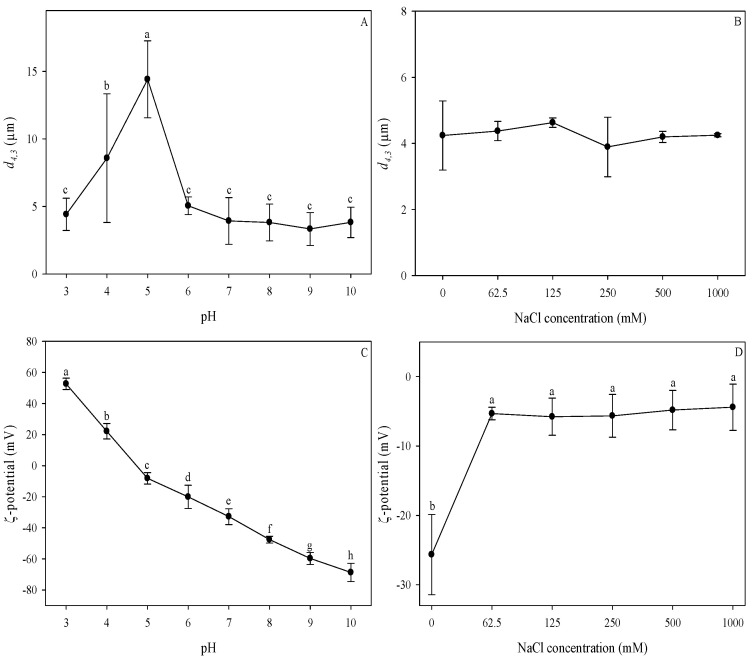
Effect of pH (**A**,**C**) and ionic strength (**B**,**D**) on the particle size (**A**,**B**) and ζ-potential (**C**,**D**) of hemp seed oil bodies. Means that do not share a letter in the same graph are statistically significant at α = 0.05. Error bars indicate standard deviations.

The influence of pH and ionic strength on the colloidal stability of oil body fractions extracted from hemp seeds was determined. The mean particle diameter (*d*_4,3_) and ζ-potential were measured in oil body dispersions at different pH (3–10) and ionic strength (0–1000 mM) conditions, and the results are shown in Figure 6. Extreme pH values (highly acidic and highly alkaline) did not affect the mean particle diameter (*d*_4,3_) of hemp seed oil bodies (Figure 6A). The particle size increased between pH 4 to 5, reaching a maximum value of 14.4 ± 2.9 μm at pH 5. However, oil bodies maintained their size that ranged from 3.3 ± 1.2 μm to 5.1 ± 0.7 μm at alkaline pH values. Particle size data also indicate oil body aggregation at pH values close to the point of zero charge. Similarly, other authors observed that the average particle size of in oil body fractions was relatively small at pH values far from the isoelectric point [14]. Aggregation of oil bodies at different pH values is dependent on electrostatic interactions: a strong electrostatic repulsion prevents the oil bodies from aggregating at pH values away from their isoelectric point [11]. On the other hand, at pH values close to the isoelectric point, there is weak electrostatic repulsion that cannot overcome the attractive forces, thereby causing oil bodies to flocculate [8,45,46]. As such, the flocculation of oil bodies at pH values close to the isoelectric point could be attributed to the weak electrostatic repulsion due to the zero net charge of the oleosins at the interface [8,25].

The effect of pH on the ζ-potential of hemp seed oil bodies is illustrated in Figure 6C. As the pH increased, the surface charge changed from positive to negative, and hemp seed oil bodies were found to be negatively charged at neutral pH. The point of zero charge was observed between pH 4 and 4.5; at pH values below this, oil bodies were positively charged, and at pH values above this, they were negatively charged. The highest ζ-potential value of −68.7 ± 5.9 mV was observed at pH 10. The effect of pH on the surface charge of the hemp seed oil body fraction is consistent with other types of oil bodies, wherein the ζ-potential changes from positive to negative with the increase in pH and had a zero value at the isoelectric point [14,46]. This behaviour of oil bodies under different pH conditions is similar to that observed in protein-stabilised emulsions, where droplets are positively charged at low pH, have zero charge at the isoelectric point, and are negatively charged at high pH [44]. Hence, it can be inferred that the membrane-specific proteins (e.g., oleosins) of oil bodies are mostly responsible for these changes in surface charge of oil bodies at various pH values [14,44]. The isoelectric point determined for hemp seed oil bodies was comparable to those reported for maize and soybean oil bodies, which were between pH 4 and 5 [11,14,46].

The changes in the particle size (*d*_4,3_) and ζ-potential of the oil bodies at different ionic strengths are shown in Figure 6B,D. Variations of the NaCl concentration in hemp seed oil bodies dispersions did not have a significant effect on the *d*_4,3_. In contrast, the increase in NaCl concentration resulted in a significant decrease in the ζ-potential of the oil bodies. After the addition of NaCl, the magnitude of the ζ-potential decreased from −25.8 ± 5.8 to around −5 mV at 62.5 to 1000 mM NaCl concentration. No significant difference was observed for the ζ-potential at all the NaCl concentrations studied. Generally, increasing the ionic strength of the solution causes a reduction of the charge density of colloidal suspensions due to electrostatic screening effects [44]. The charges are screened until a point where flocculation cannot be prevented, thereby causing the formation of aggregates. In the case of hemp seed oil bodies, a reduction in the magnitude of the ζ-potential was observed with the addition of NaCl at the lowest concentration; it remained similar as the salt concentration increased. The negative charge of oil bodies, mainly attributed to the phospholipids and oleosins at the interface, seems to be largely affected by small changes in the ionic strength causing a decrease of the negative charge density of the oil bodies interface. Similarly, other types of oil bodies such as maize, soybean, and safflower exhibited a reduction in ζ-potential with the addition of salt [11,14,47]. However, contrary to the decrease in electric potential, no significant change in the mean particle diameter was observed despite the increase in the ionic strength of the solution. The resistance of oil bodies to flocculation can be attributed to the oleosins at the interface, which not only provide electrostatic repulsion but also steric hindrance that enhances their stability [8]. This is supported by the presence of phospholipids that promote the firm anchorage of the oleosins, strengthening the oil body interface [48]. Emulsions that are sterically stabilised are more resistant to variations in pH and ionic strength compared to electrostatically stabilised emulsions [44]. Thus, the reduction in the charge density was not enough to cause the flocculation of oil bodies due to steric effects provided by the oleosins at the oil body surface.

## 4. Conclusions

Overall, this work determined the composition, structural, and physicochemical properties of oil bodies from hemp seed. The dense packing oil bodies within the cell along with other cell components showed a high degree of flexibility of hemp seed oil bodies. In aqueous solutions, hemp seed oil bodies showed a high net negative charge at neutral pH, which prevented flocculation. The CLSM images showed that the oil bodies consisted of a lipid core surrounded by an interface consisting of proteins and phospholipids. The lipids consisted of long-chain fatty acids, with linoleic acid comprising 61% of the total fatty acids and an *ω*-6/*ω*-3 ratio of 2.6:1. The fatty acid composition of polar lipids was dominated by C_16:0_, C_18:1_, C_18:2_, and C_18:3_, most likely due to the presence of phospholipids and sulfolipids in great proportions. The investigation of interfacial proteins suggests that the oleosins most likely had a molecular weight of approximately 15 kDa and did not show disulfide bonding. After isolation of oil bodies, they exhibited minimal flocculation in an aqueous suspension, as evidenced by their high ζ-potential and monomodal size distribution when dispersed in water and SDS. Hemp seed oil bodies were negatively charged at neutral pH and aggregated at pH values close to their isoelectric point, exhibiting a similar behaviour to protein-stabilised emulsions at different pH values. With the addition of salt, a reduction in ζ-potential was observed, but no further increase in particle diameter was seen with increasing salt concentration, indicating that steric effects of the oleosins at the interface were able to resist the reduction in electrical potential. Crude hemp seed oil bodies can provide a naturally stable emulsion and have the potential to be used in a variety of food applications.

## Figures and Tables

**Table 1:** Proximate composition of hemp seed oil bodies.

Parameter	Composition (%, Wet Basis)
Moisture	22.80 ± 2.09
Fat	79.30 ± 2.78
Crude protein	1.50 ± 0.22
Ash	0.20 ± 0.01

**Table 2:** Fatty acid composition (%) of lipids in hemp seed oil bodies, and their neutral and polar fractions.

Fatty Acid	Total Fat Percentage (%)		
	Total Lipids	Neutral Lipids	Polar Lipids
C_16:0_ Palmitic acid	6.10 ± 0.02	5.95 ± 0.03	8.46 ± 0.05
C_18:0_ Stearic acid	2.91 ± 0.03	2.86 ± 0.06	2.98 ± 0.07
C_18:1n9_ Oleic acid	15.10 ± 0.06	15.00 ± 0.15	13.44 ± 0.20
C_18:2n6_ Linoleic acid	60.74 ± 0.32	61.07 ± 0.20	47.36 ± 0.47
C_18:3n6_ γ-Linolenic acid	0.039 ± 0.005	0	1.23 ± 0.04
C_18:3n3_ α-Linolenic acid	11.66 ± 0.33	11.70 ± 0.23	23.62 ± 0.96
C_20:0_ Arachidic acid	1.98 ± 0.01	1.99 ± 0.06	1.59 ± 0.01
C_20:1_ Eicosenoic acid	1.00 ± 0.03	0.96 ± 0.02	0.68 ± 0.10
C_20:2_ Eicosadienoic acid	0.486 ± 0.035	0.467 ± 0.015	0.644 ± 0.082

## Data Availability

The datasets used and/or analysed during the current study are available from the corresponding author on request.
